# Somatoform dissociation and posttraumatic stress syndrome – two sides of the same medal? A comparison of symptom profiles, trauma history and altered affect regulation between patients with functional neurological symptoms and patients with PTSD

**DOI:** 10.1186/s12888-017-1414-z

**Published:** 2017-07-11

**Authors:** Johanna Kienle, Brigitte Rockstroh, Martin Bohus, Johanna Fiess, Silke Huffziger, Astrid Steffen-Klatt

**Affiliations:** 1 Department of Psychology, University of Konstanz, P.O.Box 905, D-78457 Konstanz, Germany; 2 Institute for Psychiatric and Psychosomatic Psychotherapy, Central Institute of Mental Health, Heidelberg University, J5, 68159 Mannheim, Germany; 3 Department of Health, Antwerp University, Universiteitsplein 1, 2610 Wilrijk, Belgium

**Keywords:** Dissociative disorders, Posttraumatic stress disorder, Somatoform dissociation, Functional neurological symptoms, Conversion, Traumatic life events, Alexithymia

## Abstract

**Background:**

History of traumatic experience is common in dissociative disorder (DD), and similarity of symptoms and characteristics between DD and posttraumatic stress disorder (PTSD) encouraged to consider DD as trauma-related disorder. However, conceptualization of DD as a trauma-related syndrome would critically affect diagnosis and treatment strategies. The present study addressed overlap and disparity of DD and PTSD by directly comparing correspondence of symptoms, adverse/traumatic experience, and altered affect regulation between patients diagnosed with dissociative disorder (characterized by negative functional neurological symptoms) and patients diagnosed with PTSD.

**Methods:**

Somatoform and psychoform dissociation, symptoms of posttraumatic stress, general childhood adversities and lifetime traumata, and alexithymia as index of altered affect regulation were screened with standardized questionnaires and semi-structured interviews in 60 patients with DD (ICD-codes F44.4, F44.6, F44.7), 39 patients with PTSD (ICD-code F43.1), and 40 healthy comparison participants (HC).

**Results:**

DD and PTSD patients scored higher than HC on somatoform and psychoform dissociative symptom scales and alexithymia, and reported more childhood adversities and higher trauma load. PTSD patients reported higher symptom severity and more traumata than DD patients. Those 20 DD patients who met criteria of co-occuring PTSD did not differ from PTSD patients in the amount of reported symptoms of somatoform dissociation, physical and emotional childhood adversities and lifetime traumata, while emotional neglect/abuse in childhood distinguished DD patients with and without co-occuring PTSD (DD patients with co-occuring PTSD reporting more emotional maltreatment).

**Conclusion:**

The pattern of distinctive somatoform and psychoform dissociative symptom severity, type of childhood and lifetime traumata, and amount of alexithymia suggests that DD and PTSD are distinctive syndromes and, therefore, challenges the conceptualization of DD as trauma-related disorder. Together with the detected close correspondence of symptom and experience profiles in DD patients with co-occuring PTSD and PTSD patients, these findings suggest that adverse/traumatic experience may intensify dissociative symptoms, but are not a necessary condition in the generation of functional neurological symptoms. Still, diagnosis and treatment of DD need to consider this impact of traumata and post-traumatic stress symptoms.

## Background

Dissociative disorders (DD), characterized amongst others by loss of sensations and control of bodily movements [1], are often related to traumatic experience like sexual abuse [2, 3], and emotional neglect or abuse [4, 5]. Hence, it has been discussed whether DD can be conceived of as trauma-related syndrome [6–9]. Posttraumatic stress disorder (PTSD), as a prominent representative of trauma-related disorders, is defined as response to life-threatening events (e.g. war, rape, torture or natural disaster) with symptoms like intrusions, hyperarousal and avoidance. Severe trauma, particularly sexual and here predominantly childhood sexual trauma, has been proposed as important source of somatoform and psychoform dissociation, potentially crucial in the development of DD [10–13]. Yet, somatoform dissociative symptoms have been reported in PTSD patients as well [14], despite emphasis on psychoform dissociative symptoms [15–17]. Nijenhuis introduced the concept of somatoform dissociation, referring to dissociative symptoms, that phenomenologically involve the body and comprise reduction up to complete loss of sensory perception and/or loss of motor control (negative somatoform dissociation) as well as involuntary perception of sensory (e.g. prickling), motor (e.g. tremor) and/or pain symptoms (positive somatoform dissociation) [18, 19]. On the contrary, psychoform dissociation describes a form of dissociation, that phenomenologically involves the mind [19] and pertains to disrupted mental processes such as consciousness, memory, identity and emotion, manifest in symptoms of depersonalization, derealisation, dissociative amnesia and/or out-of-body experience [17]. Often only those phenomena that Nijenhuis and other authors describe as “psychoform dissociation/dissociative symptoms” are subsumed under the label of “dissociation” or “dissociative symptoms” [17]. Beyond similarity of dissociative symptoms in PTSD and DD, the impact of dissociation in PTSD is mainly attributed to trauma severity, as peri-traumatic (mainly psychoform) dissociation and physiological components like fainting (see shut-down dissociation below) may foster later PTSD development and diagnosis [17]. Concerning somatoform dissociative symptoms in PTSD and DD, the concept of the defense cascade can explain the relation: Existential threat first prompts excessive physiological arousal (to prepare the organisms for fight/flight responses), which upon lack of escape options turns into a “shutdown” response. Fainting and immobility as manifestations of vagal dominance represent typical symptoms of such “shutdown” [14, 20–22] and can be described as somatoform dissociation, leading e.g. to functional neurological symptoms [23, 24]. Alexithymia, the deficient ability to perceive and verbally express emotions [25], signifies another correspondence between DD and PTSD. As representative of altered affect regulation alexithymia has been shown in DD patients [26–29], as well as in PTSD patients [30]. Frewen and colleagues reported positive correlations between alexithymia, PTSD symptom severity, dissociative symptom severity, and childhood abuse and neglect in PTSD patients, while Sondergaard and Theorell [31] determined evolving alexithymia as predictor of self-rated PTSD (but not depressive) symptoms in refugees. A recent study by Terock and colleagues reported alexithymia as predictor of adult psychoform dissociative symptoms independent of the effects of PTSD and childhood trauma [32]. Furthermore, alexithymia was found to predict suicidal attempt in veterans diagnosed with PTSD [33].

In the present study, symptom profiles, trauma histories and alexithymia were compared between the two diagnostic categories DD and PTSD with the hypotheses that (1) a common “trauma-related” syndrome becomes manifest in similar somatoform and psychoform dissociative symptoms and similar trauma histories across the diagnostic groups; (2) the relation of trauma history to symptom expression indicates a common meaning of trauma in the generation of DD and PTSD; (3) correspondence of alexithymia between the two diagnostic groups and its relation to dissociative symptom expression indicates the important role of affect regulation in the development of DD and PTSD.

In the present sample, patients with ICD-10 diagnoses of DD were characterized by dissociative motor disorder, i.e. “loss of ability to move the whole or part of a limb or limbs” (ICD-10, p. 127), dissociative anaesthesia and sensory loss, referring to impaired tactile, auditory or sensory perception, or mixed dissociative disorder integrating both.^1^ Patients with ICD-10 diagnosis of PTSD represented trauma-related disorders. Matching symptom profiles, trauma history, and alexithymia in the two diagnostic groups should indicate the correspondence of syndromes, thus clarifying the conception of DD as trauma-related disorder (thereby informing the meaning of trauma in the generation of DD and supporting attuned diagnostics and treatment strategies).

## Methods

### Participants

Sixty patients^2^ with ICD-10 diagnoses of dissociative disorder (DD; ICD-10 codes F44.4, F44.6, F44.7), 39 patients with an ICD-10 diagnosis of posttraumatic stress disorder (PTSD; ICD-10 code F43.1), and 40 healthy comparison participants (HC) participated in the study. DD patients were recruited at the local neurological rehabilitation centre (Kliniken Schmieder Konstanz and Gailingen). Following neurological routine, inclusion criteria were at least one core negative somatoform dissociative symptom. Exclusion criteria were central nervous lesions and positive somatoform dissociative symptoms (e.g. seizures). Similar subtypes of dissociative disorders, characterized by negative somatoform dissociative symptoms were selected in order to assure homogeneity of the study sample. Diagnoses were given by at least two experienced psychiatrics and neurologists. Patients diagnosed with PTSD were recruited at the Department of Psychosomatic Medicine and Psychotherapy of the Central Institute for Mental Health (CIMH, Mannheim). Diagnoses were based on DSM-IV criteria (Structured clinical interview for DSM-IV and International Personality Disorder Examination [34, 35]). Comorbid conditions are summarized in Table 5. HC were recruited from the local community by flyer and oral advertisement and selected to be comparable to the patient samples with respect to age and gender distribution. HC were screened for DSM-axis I and II diagnoses using the German version of the MINI international Neuropsychiatric Interview [36]. Volunteers who reported any kind of current or past neurological or mental disorders or the use of psychoactive medication were not included in the sample. Table 1 summarizes demographic information of the three groups. While groups did not differ in gender and age distribution, HC had a higher educational level than patients with DD and PTSD.

**Table 1:** Sociodemographic information of study samples

	DD patients	PTSD patients	HC	Comparison across groups
*N*	60	39	40	
Gender (f/m)	45/15	33/6	34/6	χ2 = 2.09, *p* = .35
Age (M ± SD)	42.6 ± 12.31	41.3 ± 9.32	40.6 ± 11.9	*H*(2) = 0.9, *p* = .64
Years schooling (M ± SD)	10.8 ± 2.27	10.7 ± 1.59	11.8 ± 1.49	*H*(2) = 11.69, *p* = .003

*Note*. *DD* dissociative disorder, *PTSD* posttraumatic stress disorder, *HC* healthy comparison participants, *f* female, *m* male

### Design and procedure

The study design was approved by the ethics committee of the University of Konstanz, the board of the neurological rehabilitation centre Kliniken Schmieder and the board of Mannheim medical faculty of Heidelberg University. Prior to data assessment, participants were informed about the study purpose and the procedures and signed written informed consent. Thereafter, childhood adversities and lifetime traumata were assessed using standardized semi-structured interviews administered by trained project members. Each interview lasted about 1.5 h. In addition, dissociation, PTSD symptoms and alexithymia were screened with questionnaires, which participants filled in on their own (project members being available for questions). Data assessment was accomplished at the institution of recruitment and lasted altogether about 2-3 h per participant. HC and PTSD patients at the CIMH received a bonus of 20 Euro for participation, while DD patients filled in the questionnaires/interview set as part of their treatment.

### Material

Somatoform *dissociative symptoms* were measured with the Somatoform Dissociation Questionnaire (SDQ-20 [18]; German version by [37], see appendix for details).^3^ Psychoform dissociation was assessed with the Dissociative Experience Scale (DES [38]; German version by [39]).^4^ Both scales, SDQ-20 and DES, show good internal consistency and reliability (SDQ-20, α = .914, *r*_*tt*_ = .89, [37], DES, α = .94, *r*_*tt*_ = .82, [38]). Severity of *PTSD symptoms* (hyperarousal, intrusions, avoidance) and number of *lifetime traumatic experience* were verified with the Posttraumatic Stress Diagnostic Scale (PDS [40])^5^ which shows good internal consistency (α = .94) and validity [41]. *Adverse experience in childhood and adolescence* were assessed using the German version KERF (‘Kindheitserfahrungen’, [42]) of the Maltreatment and Abuse Chronology of Exposure [43].^6^ As measure of altered affect regulation, alexithymia was assessed with the Toronto Alexithymia Scale (TAS-26 [44]; German version by [45], internal consistency α = .84).^7^

### Data analysis

Measures of symptom severity, adverse/traumatic experience, and alexithymia were first compared between the three samples (DD, PTSD, HC). Per PDS, 20 of the 60 patients diagnosed with DD met criteria of co-occuring PTSD. Therefore, analyses were repeated for four subgroups: patients with DD and co-occuring PTSD (DD^+^), patients with DD without co-occuring PTSD (DD^−^), patients with PTSD, and HC. The four groups did not differ in gender (χ2 (3, *N* = 139) = 4.01, *p* = .26) and age distribution (*H*(3) = .91, *p* = .82), while the significant difference in education between HC and the three patient groups remained.

Since data within subgroups was not normally distributed, with positive skew in HC and negative skew in both patient groups and the assumption of homogeneity of variance was not met, we applied non-parametric testing of group differences using the Kruskall-Wallis test. Post-hoc subgroup differences were verified by Mann-Whitney tests Bonferroni-corrected for multiple comparisons with alpha corrected to .007. Effect sizes were calculated using the estimate “r” described by Rosenthal ﻿(1991) which is robust to unequal sample sizes [46].

The impact of childhood adversities and traumata on somatoform symptom severity was examined by forced entry multiple regression analyses including overall childhood severity (KERF_Sum) or the number of lifetime traumata (PDS_Event) and PTSD symptom severity (PDS_Sym) as predictors of somatoform dissociative symptom severity (SDQ-20).

## Results

### Symptom severity across groups (HC vs. DD vs. PTSD)

PTSD patients scored higher on dissociative symptom scales and on the posttraumatic stress symptom scale than DD patients and HC (see Table 2 for mean scores and Table 3 for statistical group differences). The comparison of the four subgroups (DD^+^ vs. DD^−^ vs. PTSD vs. HC; see Table 4) confirmed different somatoform dissociation and symptoms of posttraumatic stress between subgroups except for the comparison between DD^+^ and PTSD patients (see Table 4). This indicates symptom correspondence between these two subgroups, although DD^+^ expressed less psychoform dissociation than PTSD patients.

**Table 2:** Median and range of symptom severity, adversity/trauma measures and alexithymia scoresok

	HC *n* = 40	DD^−^ *n* = 40	DD^+^ *n* = 20	PTSD patients *n* = 39
	median *(range)*	median *(range)*	median *(range)*	median *(range)*
Symptom severity				
SDQ-20	21 *(20 – 26)* *n* = 40	28.5 *(20 – 54)* *n* = 40	36.5 *(29 – 56)* *n* = 18	36 *(20 – 91)* *n* = 39
DES	6.49 *(0 – 25.36)* *n* = 40	12.14 *(0 – 48.57)* *n* = 40	19.64 *(9.64 – 51.79)* *n* = 20	38.57 *(8.57 – 80.71)* *n* = 39
PDS_Sym	0 *(0 – 8)* *n* = 40	4 *(0 – 38)* *n* = 40	30 *(16 – 46)* *n* = 20	36 *(13 – 48)* *n* = 39
Adversity/trauma measures				
KERF_Sum	35.08 *(0 – 235.5)* *n* = 40	90.17 *(0 – 533.08)* *n* = 38	219.67 *(18.83 – 605)* *n* = 17	369 *(60 – 885.75)* *n* = 39
KERF_Phy	7.33 *(0 – 141.67)* *n* = 40	40.33 *(0 – 229.3)* *n* = 38	33 *(1.67 – 304)* *n* = 17	141 *(0 – 462)* *n* = 39
KERF_Emo	25 *(0 – 218)* *n* = 40	52.5 *(0 – 329)* *n* = 38	141 *(14.5 – 362)* *n* = 17	239.5 *(51 – 423.5)* *n* = 39
KERF_Sex	0 *(0 – 1.3)* *n* = 40	0 *(0 – 23.8)* *n* = 38	0 *(0 – 27.5)* *n* = 17	7.5 *(0 – 40)* *n* = 39
PDS_Event	1.5 *(0 – 5)* *n* = 40	3 *(0 – 7)* *n* = 40	4 *(2 – 7*) *n* = 20	5 *(2 – 8)* *n* = 39
Alexithymia				
TAS-26	1.94 *(1.33 – 3.05)* *n* = 40	2.58 *(1.39 – 3.44)* *n* = 40	2.92 *(1.83 – 4.33)* *n* = 20	3.44 *(1.66 – 4.55)* *n* = 39

*Note*. *HC* healthy comparison participants, *DD* ^*−*^ patients diagnosed with dissociative disorder without co-occuring PTSD, *DD* ^*+*^ patients diagnosed with dissociative disorder with co-occuring PTSD, *PTSD* posttraumatic stress disorder, *SDQ-20* severity of somatoform dissociative symptoms, verified by the *Somatoform Dissociation Questionnaire*, *DES* severity of psychoform dissociative symptoms, using the *Dissociative Experience Scale*, *PDS_Sym* load of posttraumatic symptoms, *KERF_Sum* overall exposure to childhood adversities, *KERF_Phy* physical maltreatment during childhood, *KERF_Emo* emotional neglect and maltreatment during childhood, *KERF_Sex* sexual violence during childhood, *PDS_Event* Sum of lifetime traumatic experience assessed with the *Posttraumatic Diagnostic Scale*, *TAS-26* Alexithymia, assessed with the *Toronto Alexithymia Scale*

**Table 3:** Group comparisons (HC, DD, PTSD) – inferential statistics of symptom severity, adversity/trauma measures and alexithymia scores

	Comparison across groups	DD patients vs. HC *n* = 100	PTSD patients vs. HC *n* = 79	DD patients vs. PTSD patients *n* = 99
Symptom severity				
SDQ-20	*H*(2) = 70.62 *p* < .001	*U* = 172.5, *z* = −7.17 *p* < .001 *r* = −0.72	*U* = 62, *z* = −7.07 *p* < .001 *r* = −0.80	*U* = 727, *z* = −2.98 *p* = .021 *r* = −0.30
DES	*H*(2) = 68.77 *p* < .001	*U* = 585, *z* = −4.33 *p* < .001 *r* = −0.43	*U* = 24.5, *z* = −7.41 *p* < .001 *r* = −0.83	*U* = 348, *z* = −5.89 *p* < .001 *r* = −0.59
PDS_Sym	*H*(2) = 82.23 *p* < .001	*U* = 402.5, *z* = −5.80 *p* < .001 *r* = −0.58	*U* = 0, *z* = −7.8 *p* < .001 *r* = −0.88	*U* = 322, *z* = −6.08 *p* < .001 *r* = −0.61
Adversity/trauma measures				
KERF_Sum	*H*(2) = 57.55 *p* < .001	*U* = 610, *p* = −3.70 *p* < .001 *r* = −0.37	*U* = 49, *z* = −7.05 *p* < .001 *r* = −0.79	*U* = 364.5, *z* = −5.20 *p* < .001 *r* = −0.52
KERF_Phy	*H*(2) = 49.07 *p* < .001	*U* = 647.5, *z* = −3.43 *p* = .001 *r* = −0.34	*U* = 113.5, *p* = −6.55 *p* < .001 *r* = −0.73	*U* = 460, *z* = −4.70 *p* < .001 *r* = −0.47
KERF_Emo	*H*(2) = 60.12 *p* < .001	*U* = 606, *z* = −3.72 *p* < .001 *r* = −0.37	*U* = 47.5, *z* = −7.19 *p* < .001 *r* = −0.80	*U* = 377.5, *z* = −5.33 *p* < .001 *r* = −0.53
KERF_Sex	*H*(2) = 52.83 *p* < .001	*U* = 943.5, *z* = −2.22 n.s.	*U* = 226, *z* = −6.29 *p* < .001 *r* = −0.70	*U* = 472.5, *z* = −5.22 *p* < .001 *r* = −0.52
PDS_Event	*H*(2) = 49.32 *p* < .001	*U* = 536, *z* = −4.73 *p* < .001 *r* = −0.47	*U* = 108.5, *z* = −6.66 *p* < .001 *r* = −0.74	*U* = 708.5, *z* = −3.36 *p* = .007 *r* = −0.34
Alexithymia				
TAS-26	*H*(2) = 70.34 *p* < .001	*U* = 387, *z* = −5.72 *p* < .001 *r* = −0.57	*U* = 67.5, *z* = −6.99 *p* < .001 *r* = −0.78	*U* = 408, *z* = −5.46 *p* < .001 *r* = −0.54

*Note*. *HC* healthy comparison participants, *DD* patients diagnosed with dissociative disorder, *PTSD* posttraumatic stress disorder, *SDQ-20* severity of somatoform dissociative symptoms, verified by the *Somatoform Dissociation Questionnaire*, *DES* severity of psychoform dissociative symptoms, using the *Dissociative Experience Scale*, *PDS_Sym* load of posttraumatic symptoms, *KERF_Sum* overall exposure to childhood adversities, *KERF_Phy* physical maltreatment during childhood, *KERF_Emo* emotional neglect and maltreatment during childhood, *KERF_Sex* sexual violence during childhood, *PDS_Event* Sum of lifetime traumatic experience assessed with the *Posttraumatic Diagnostic Scale*, *TAS-26* Alexithymia, assessed with the *Toronto Alexithymia Scale*. Effect sizes were calculated using the estimate “r” described by Rosenthal, 1991 [46]

**Table 4:** Group comparisons (HC, DD^+^, DD^−^, PTSD) – inferential statistics of symptom severity, adversity/trauma measures and alexithymia scores

	Comparison across groups	DD^+^ vs. PTSD patients *n* = 59	DD^−^ vs. PTSD patients *n* = 79	DD^−^ vs. HC *n* = 80	DD^+^ vs. DD^−^ *n* = 60
Symptom severity					
SDQ	*H*(3) = 77.61 *p* < .001	*U* = 332.5, *z* = −3.20 *n.s.*	*U* = 394.5, *z* = −3.78 *p* < .001 *r* = −0.41	*U* = 172.5, *z* = −6.08 *p* < .001 *r* = −0.68	*U* = 151.5, *z* = −3.51 *p* < .001 *r* = −0.45
DES	*H*(3) = 78.20 *p* < .001	*U* = 197.5, *z* = −3.08 *p* = .014 *r* = −0.40	*U* = 150.5, *z* = −6.17 *p* < .001 *r* = −0.61	*U* = 528.5, *z* = −2.61 *n.s.*	*U* = 168.5, *z* = −3.63 *p* < .001 *r* = −0.47
PDS_Sym	*H*(3) = 102.77 *p* < .001	*U* = 267.5, *z* = −1.96 *n.s.*	*U* = 54.5, *z* = −7.13 *p* < .001 *r* = −0.8	*U* = 402.5, *z* = −4.09 *p* < .001 *r* = −0.46	*U* = 37, *z* = −5.72 *p* < .001 *r* = −0.74
Adversity/trauma measures					
KERF_Sum	*H*(3) = 61.63 *p* < .001	*U* = 169, *z* = −2.71 *p* = .049 *r* = −0.35	*U* = 195.5, *z* = −5.38 *p* < .001 *r* = −0.61	*U* = 510, *z* = −2.5 n.s.	*U* = 203, *z* = −2.19 n.s.
KERF_Phy	*H*(3) = 49.98 *p* < .001	*U* = 183, *z* = −2.65 *n.s.*	*U* = 277, *z* = −4.73 *p* < .001 *r* = −0.53	*U* = 481.5, *z* = −2.8 *p* = 0.03 *r* = −0.31	*U* = 271, *z* = −0.95 *n.s.*
KERF_Emo	*H*(3) = 66.10 *p* < .001	*U* = 194.5, *z* = −2.44 *n.s.*	*U* = 183, *z* = −5.69 *p* < .001 *r* = −0.63	*U* = 512.5, *z* = −2.47 *n.s.*	*U* = 169.5, *z* = −2.80 *p* = .035 *r* = −0.36
Mace_Sex	*H*(3) = 53.50 *p* < .001	*U* = 173, *z* = −2.94 *p* = .021 *r* = −0.38	*U* = 299.5, *z* = 4.99 *p* < .001 *r* = −0.56	*U* = 676.5, *z* = −1.81 *n.s.*	*U* = 287.5, *z* = −1.00 *n.s.*
PDS_Event	*H*(3) = 52.07 *p* < .001	*U* = 301.5, *z* = −1.45 *n.s.*	*U* = 407, *z* = −3.72 *p* < .001 *r* = −0.42	*U* = 424.5, *z* = 3.67 *p* < .001 *r* = −0.41	*U* = 291.5, *z* = −1.73 *n.s.*
Alexithymia					
TAS-26	*H*(3) = 74.97 *p* < .001	*U* = 211, *z* = −2.87 *p* = .028 *r* = −0.37	*U* = 197, *z* = −5.72 *p* < .001 *r* = −0.64	*U* = 325.0, *z* = 4.58 *p* < .001 *r* = −0.51	*U* = 226, *z* = −2.73 *p* = 0.042 *r* = −0.35

*Note*. *HC* healthy comparison participants, *DD* ^*−*^ patients diagnosed with dissociative disorder without co-occuring PTSD, *DD* ^*+*^ patients diagnosed with dissociative disorder with co-occuring PTSD, *PTSD* posttraumatic stress disorder, *SDQ-20* severity of somatoform dissociative symptoms, verified by the *Somatoform Dissociation Questionnaire*, *DES* severity of psychoform dissociative symptoms, using the *Dissociative Experience Scale*, *PDS_Sym* load of posttraumatic symptoms, *KERF_Sum* overall exposure to childhood adversities, *KERF_Phy* physical maltreatment during childhood, *KERF_Emo* emotional neglect and maltreatment during childhood, *KERF_Sex* sexual violence during childhood, *PDS_Event* Sum of lifetime traumatic experience assessed with the *Posttraumatic Diagnostic Scale*, *TAS-26* Alexithymia, assessed with the *Toronto Alexithymia Scale*. Effect sizes were calculated using the estimate “r” described by Rosenthal, 1991 [46]

### Adversity and trauma measures across groups (HC vs. DD vs PTSD)

PTSD patients reported more traumatic events across lifetime than the other groups (median see Tables 2 and 3). PTSD patients had also experienced more childhood adversities (KERF_Sum) than DD and HC. Differences between DD patients and PTSD patients were confirmed for physical, emotional and sexual maltreatment in childhood (see Table 3). Importantly, physical and emotional maltreatment, and lifetime traumata did not differ between DD^+^ and PTSD patients, whereas PTSD patients reported more sexual abuse than DD^+^. Emotional neglect/abuse in childhood distinguished DD^+^ and DD^−^ (DD^+^ reported more emotional maltreatment than DD^−^; see Table 4). DD patients reported more overall exposure to childhood adversities than HC, however there was no significant difference between the subgroup of DD patients without co-occuring PTSD and HC. Except for physical maltreatment, childhood adversities did not differ between DD^−^ and HC.

### Relationship between trauma/maltreatment and symptom severity (overall and within subgroups)

**Fig. 1:**
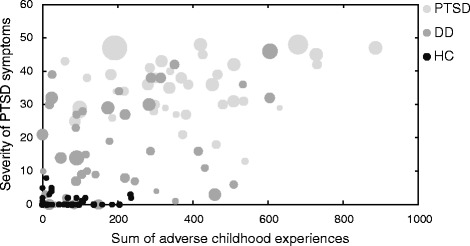
Relationship between the sum of adverse childhood experience (abscissa), severity of PTSD symptoms (ordinate), and somatoform dissociation (SDQ-20 scores), expressed by size of filled circles: larger filled circles correspond to higher SDQ-scores. Each circle represents a participant; subgroups are reflected by color-coding with *light grey circles* representing PTSD patients, *dark grey circles* DD patients, *black circles* HC

**Fig. 2:**
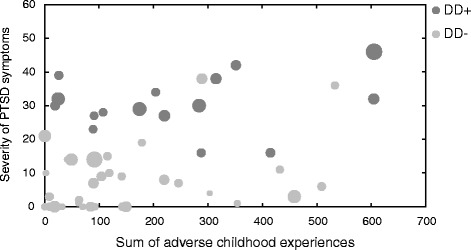
Relationship between the sum of adverse childhood experience (abscissa), severity of PTSD symptoms (ordinate), and somatoform dissociation (expressed by size of filled circles: larger filled circles correspond to higher SDQ-scores) illustrated separately for DD patients with (DD^+^; *dark grey circles*) and DD patients without (DD^−^; *light grey circles*) co-occuring PTSD diagnosis

The relationship between the sum of adverse childhood experience, posttraumatic symptom severity, and somatoform dissociative symptom severity is illustrated for the entire sample (HC, DD, PTSD) in Fig. 1 and for the two subsamples of DD patients (DD^+^ and DD^−^) in Fig. 2.

**Fig. 3:**
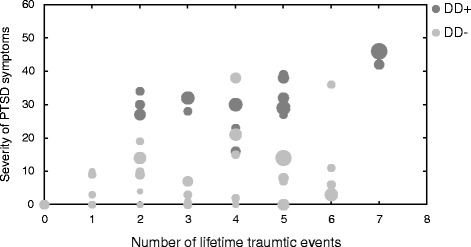
Relationship between the number of lifetime traumatic events (abscissa), severity of PTSD symptoms (ordinate), and somatoform dissociation (expressed by size of filled circles: larger filled circles correspond to higher SDQ-scores) separately for DD patients with (DD^+^; *dark grey circles*) and DD patients without (DD^−^; *light grey circles*) co-occuring PTSD diagnosis

Figure 3 shows the association between the number of lifetime traumatic events, posttraumatic symptom severity, and somatoform dissociation for the two subsamples of DD patients.

Figures 2 and 3 suggest a relationship between PTSD symptoms and severity of somatoform dissociation in DD patients with low and with high number of adverse childhood experience and traumata. Multiple regression analysis confirmed that adverse childhood experience, number of lifetime traumatic events, and severity of PTSD symptoms did explain 30% of variance of somatoform dissociation in DD patients (*R*2 = .30, *F*(3,53) = 7.25, *p* < .001). However, adverse childhood experience (β = −.002, *p* = .99) and number of lifetime traumatic events (β = .27, *p* = .08) did not explain variance in addition to severity of PTSD symptoms (β = .38, *p* < .001). This relationship was also observed in patients with PTSD: the three factors did account for 31% of variation in somatoform dissociation (*R*2 = .31, *F*(3,36) = 5.11, *p* < .01). Again, adverse childhood experience (β = .075, *p* = .63) and number of liefetime traumatic events (β = .055, *p* = .71) did not explain variance in addition to severity of PTSD symptoms (β = .52, *p* < .001).

### Alexithymia across groups (HC vs. DD vs. PTSD)

PTSD patients scored higher on the alexithymia scale (TAS-26) than DD patients, and both patient groups expressed more alexithymia than HC (see Table 2). Although alexithymia scores in the present DD sample were lower than expected from the literature, alexithymia was related to dissociative and posttraumatic stress symptom severity in DD patients, in that more intense alexithymia varied with more intense somatoform dissociative (*r* = .30, *p* = .02), psychoform dissociative (*r* = .40, *p* = .001), and PTSD symptoms (*r* = .31, *p* = .02). For patients with PTSD, alexithymia was also positively related to somatoform dissociative symptoms (*r* = .34, *p* = .03).

## Discussion

A history of traumatic experience and corresponding symptoms in affected patients have encouraged the association of dissociative and posttraumatic stress disorders. However, conceiving of both diagnoses as the same syndrome asks for conformity on all levels. The present comparison of characteristic symptoms, trauma and maltreatment history between patients diagnosed with DD or PTSD (per hypothesis 1) demonstrated group differences in symptom prominence (e.g., psychoform vs. somatoform symptoms) and trauma profiles (e.g. emotional vs. sexual abuse) that challenge a global assignment of DD to the category of trauma-related disorders. Rather, the conformity of DD patients with co-occuring PTSD and PTSD patients on several measures (number of lifetime traumata, amount of physical and emotional abuse in childhood, PTSD symptom severity and severity of somatoform dissociation) suggests the portrayal of a “trauma-related DD syndrome”. While accumulated traumatic experience may add to symptom severity, they are not critical for the generation of DD ﻿(compare also research by Stone and colleagues [47]). Distinction of syndromes per symptom and trauma profile does not render the association between trauma and dissociation obsolete (hypothesis 2). Indeed, the variation of severity of posttraumatic stress symptoms and dissociative symptoms with traumata and childhood maltreatment, illustrated in Figs. 1, 2 and 3, suggests an impact of coping with such experience on augmented somatoform dissociation and the development of DD. Moreover, chronic dissociative symptoms in DD and PTSD may be explained as a conditioned response upon repeated adverse experience [14, 20–22]. It is conceivable, that the individual post-traumatic learning and coping history shapes development and type of dissociative symptom (psychoform or somatoform) upon later confrontation with adverse and traumatic events [20]. For instance a learning history of early coping with the emotional consequences of trauma and maltreatment with somatic symptoms and somatoform dissociation may favour the development of DD. Alexithymia as an expression of altered emotion processing was expected to be increased in patient groups and related to symptom expression. The present diagnostic groups differed in alexithymia, in that DD patients had lower TAS scores than PTSD patients. This suggests that DD patients were able to perceive and express their emotions, although less efficiently than HC. In line with the hypothesis of modulation by learning history, and the literature [29], the positive relation between alexithymia and somatoform symptom severity might reflect the learned attribution of feelings to somatic sensations. A further factor to explain the evolution of trauma-related and dissociation-related disorders (PTSD and DD in the present example) might be the dose: The coincidence of higher trauma load (childhood adversities and lifetime traumata) and higher symptom scores in PTSD patients and DD patients with comorbid PTSD may indicate a “dose effect”, i.e., higher trauma load results in the more severe disorder as characterized by comorbidity and symptom severity [48]. Commonly, higher trauma load in patients with PTSD as well as in DD patients is described to be related to more sexual traumata. In contrast, in the present data this relation was replicated for the PTSD group only, while both DD groups report similar sexual traumata as HC. Interestingly, emotional adverse experience do differentiate between both DD groups, which may point towards emotional neglect/abuse as a distinguishing factor. Potential influe﻿nces of comorbid conditions also have to be taken into account here: Depression is a frequent comorbid disorder in PTSD [49] and DD [50] also in the present groups (Table 5). Recent research found evidence for a depressive subtype of PTSD that is associated with greater dissociative experience [51]. Since 38.5% of the current sample of PTSD patients suffered from a comorbid depressive disorder or depressive episode, this may explain the high amount of dissociative symptoms. Frequency of comorbid depressive and anxiety disorders (identified as further comorbid conditions across groups) did not significantly differ between the current patient groups, so that a major impact of this comorbidity on the between-group differences of interest seems unlikely. Current group-specific comorbidities, i.e. comorbid somatoform disorder in DD patients and comorbid borderline personality disorder in PTSD patients are comparable to results from previous studies [3, 52–54] and indicate common contributions to dissociative symptoms. Dissociative disorders are characterized by somatoform dissociative symptoms independent of comorbid PTSD diagnosis – as reflected by the amount of comorbid somatoform disorder diagnoses, which was significantly higher in patients diagnosed with DD compared to patients diagnosed with PTSD.

**Table 5:** Comorbid conditions across groups

	DD^−^ *n* = 40	DD^+^ *n* = 20	PTSD patients *n* = 39
Recurrent depressive disorder or depressive episode	9 (22.5%)	5 (25%)	15 (38.5%)
(Phobic) anxiety disorder	1 (2.5%)	4 (20%)	6 (15.4%)
Emotionally unstable personality disorder	1 (2.5%)	0 (0%)	17 (43.6%)
Somatoform disorder	15 (37.5%)	5 (25%)	3 (7.7%)

*Note*. *DD* ^*−*^ patients diagnosed with dissociative disorder without co-occuring PTSD, *DD* ^*+*^ patients diagnosed with dissociative disorder with co-occuring PTSD, *PTSD* posttraumatic stress disorder

Limitations of the present study have to be noted: (1) Different syndromes of PTSD and DD were concluded from different symptom profiles. However, as patient samples were recruited in different institutions, differences in treatment settings between the two patient groups are likely. It cannot be ruled out, that these differences may have influenced the results. (2) Many DD patients showed substantial signs of severe somatoform dissociative symptoms (like sitting in a wheelchair) parallel to low self-evaluation of somatoform dissociation (lower SDQ-20 scores than PTSD patients). This suggests, that the SDQ-20 may not properly mirror severity of functional neurological symptoms in DD patients. For each symptom the SDQ-20 evaluated the frequency of experience, while symptom duration was not assessed. Somatoform dissociative symptoms are long lasting or permanent in DD, while they may last for minutes or hours in PTSD. Thus, including symptom duration in the measurement of dissociative symptoms seems mandatory for the specification of DD syndrome and its distinction from PTSD. (3) Involving PTSD as an example of trauma-related syndrome in the present study does not justify the generalization of the present results and conclusions. Further studies should consider other trauma-related syndromes, such as acute stress disorder, adjustment disorder etc. However, complex PTSD was chosen as an example for trauma-related disorders in the present study, since it is associated with high levels of dissociation – which is especially documented in manifold studies reporting psychoform dissociative symptoms in PTSD patients [15–17] and multiple trauma experience in patients with conversion or other dissociative disorders [12, 23, 55].

## Conclusion

The present comparison of symptoms and trauma history between DD and PTSD revealed a clear distinction between the diagnostic groups, disconfirming hypothesis (1) of a common syndrome. Still, results indicate an important role of adverse/traumatic experiences and the experience of posttraumatic stress symptoms in the development of dissociative symptoms (per hypothesis 2). This specification matches the diagnostic descriptions in DSM-V, in that a relevant psychological stressor preceding the onset of functional neurological symptom disorder, earlier required as diagnostic criterion, is now labelled as a specification feature. Moreover, distinct sub-groups of DD patients with and without PTSD are also reflected in the specification of a dissociative subtype of PTSD in DSM-5 [56]. The results have important clinical implications: Adopting a context of linking dissociative and trauma-related disorders asks to consider a broad range of dissociative symptoms, not only psychoform derealization or depersonalization phenomena but also somatoform dissociative symptoms [14, 20, 21, 57]. Present results further direct attention to individual maltreatment and coping/learning history to be considered in diagnostics and treatment: While the number of traumatic events may determine the severity of distress, the individual coping history with adversities and traumata may modulate, how symptoms develop and accentuate in patients diagnosed﻿ with DD, and potentially overlap with those of individuals diagnosed with PTSD. This advocates the careful assessment of trauma history and its consequences in diagnostics and treatment of DD.

## Abbreviations

CIMH: Central institute for mental health
DD: Dissociative disorder
DD^−^: Dissociative disorder without co-occuring PTSD
DD^+^: Dissociative disorder with co-occuring PTSD
DES: Dissociative experience scale
DSM: Diagnostic and statistical manual of mental disorders
HC: Healthy comparison subject
ICD: International statistical classification of diseases and related health problems
KERF_Emo: Emotional neglect and maltreatment during childhood
KERF_Phy: Physical maltreatment during childhood
KERF_Sex: Sexual violence during childhood
KERF_Sum: Sum of adverse childhood experience
PDS: Posttraumatic symptom scale
PDS_Event: Sum of lifetime traumatic events
PDS_Sym: Load of posttraumatic stress symptoms
PTSD: Posttraumatic stress disorder
SDQ: Somatoform dissociation questionnaire
TAS: Toronto alexithymia scale

## Footnotes

1 In DSM-V these subtypes of dissociative disorder are part of the diagnosis of conversion disorder or functional neurological symptom disorder (FNSD).

2 The present sample partially overlaps with the one reported in [29].

3 The 20-item self-report instrument assesses the frequency of somatoform dissociation experienced in the preceding 1 months. It includes negative symptoms of dissociation like sensory losses and loss of motor control as well as positive symptoms of dissociation like alterations of vision, audition, taste and smell. Items are evaluated on a 5-point Likert Scale (from “This applies to me not at all” to “This applies to me extremely”), resulting in possible sum-scores between 20 and 100.

4 The DES is a 28-item self-report measure covering the domains amnesia, absorption and derealisation/depersonalisation. The percentage of experienced symptoms across lifetime is evaluated on a continuum between 0% (“never”) to 100% (“always”). Mean values are ranging from 0 to 100.

5 The PDS-symptom scale comprises 17 items, severity of each symptom being scored on a scale from 0 to 3, resulting in sum-scores between 0 and 51.

6 The KERF includes 70 items covering ten domains of experience (parental verbal abuse, parental non-verbal emotional abuse, parental physical abuse, emotional neglect, physical neglect, familial and non-familial sexual abuse, witnessed physical violence towards parents, witnessed violence towards siblings, peer emotional and peer physical violence), together with age of onset and duration of the respective experience up to age 18. The following analyses include all experience until individual onset of puberty, determined by first menarche/puberty vocal change. Convergent validity of the KERF was verified by correlation with the Childhood Trauma Questionnaire and good test-retest reliability was established (r_tt_ = .91 at 10 weeks [42]). For the present analyses sum-scores of subscales were collapsed to three broader categories: emotional abuse/neglect (KERF_Emo) with a range from 0 to 720 (including parental verbal abuse, parental non-verbal emotional abuse, emotional neglect, peer emotional violence), physical abuse/neglect (KERF_Phy) with a range from 0 to 900 (including parental physical abuse and neglect, witnessed physical violence towards parents, witnessed violence towards siblings, peer physical violence) and sexual violence (KERF_Sex) with a range from 0 to 180 (including familial and non-familial sexual abuse). In addition, the overall severity of exposure to childhood adversities until individual onset of puberty (KERF_Sum), ranging from 0 (“no childhood adversities at all”) to 1800 (“maximal exposure to all types of childhood adversities”) was calculated.

7 The 26-item self-report questionnaire assesses alexithymia on three dimensions: “difficulty identifying feelings”, “difficulty describing feelings” and “externally oriented thinking”. Overall mean values, ranging from 1 to 5 are reported.
